# Bioinformatics-Aided Venomics

**DOI:** 10.3390/toxins7062159

**Published:** 2015-06-11

**Authors:** Quentin Kaas, David J. Craik

**Affiliations:** Institute for Molecular Bioscience, the University of Queensland, Brisbane, QLD 4072, Australia; E-Mail: d.craik@imb.uq.edu.au

**Keywords:** toxins, databases, algorithms, proteomics, transcriptomics, phylogeny, molecular modeling

## Abstract

Venomics is a modern approach that combines transcriptomics and proteomics to explore the toxin content of venoms. This review will give an overview of computational approaches that have been created to classify and consolidate venomics data, as well as algorithms that have helped discovery and analysis of toxin nucleic acid and protein sequences, toxin three-dimensional structures and toxin functions. Bioinformatics is used to tackle specific challenges associated with the identification and annotations of toxins. Recognizing toxin transcript sequences among second generation sequencing data cannot rely only on basic sequence similarity because toxins are highly divergent. Mass spectrometry sequencing of mature toxins is challenging because toxins can display a large number of post-translational modifications. Identifying the mature toxin region in toxin precursor sequences requires the prediction of the cleavage sites of proprotein convertases, most of which are unknown or not well characterized. Tracing the evolutionary relationships between toxins should consider specific mechanisms of rapid evolution as well as interactions between predatory animals and prey. Rapidly determining the activity of toxins is the main bottleneck in venomics discovery, but some recent bioinformatics and molecular modeling approaches give hope that accurate predictions of toxin specificity could be made in the near future.

## 1. Introduction

Animals that use venoms for killing or paralyzing prey or for defense purposes are widespread in diverse phyla of the animal kingdom. They are found in nearly all major animal subgroups, including vertebrates (e.g., snakes, lizards, fish, toads, frogs, vampire bats, platypus, echidna), mollusks (e.g., marine cone snails, terebrids, cuttlefish, octopus, squids), arthropods (e.g., scorpions, spiders, centipedes, ticks, ants, wasps, bees), annelids (e.g., leeches), sponges and cnidarian (e.g., jellyfish, sea anemones). Animal venoms are a complex mixture of substances, mainly composed of proteins and peptides, but also containing lipids, amines and other small molecules [1]. Venom peptides have sparked particular interest because of their activity on the nervous system (blockers or agonists of ion channels, transporters or receptors) or on membranes (antibacterial, hemolytic). Larger venom proteins have enzymatic activity (phospholipase A2, proteases, and oxidases) or modify important physiological process such as coagulation (lectins). The venom of a single species can contain hundreds to several thousands of active peptides [2,3], and the total pool of unique active peptides in venoms is therefore extremely large, probably greater than ten million. For example, hundreds of thousands of different active peptides have been proposed to exist in the venoms of the greater than 700 marine cone snail species [3,4,5]. Other species extensively studied for their venom content belong to more highly diversified orders or sub-orders; for example, there are ~45,400 species of spiders [6], ~3500 species of snakes [7,8] and ~2000 species of scorpions [9].

Animal venoms are one of the richest natural sources of active compounds and have numerous pharmacological and medical applications [10]. Toxins are used as pharmaceutical tools (e.g., to characterize ion channels), and also in pharmaceutical applications (e.g., drugs) or biotechnological applications (e.g., insecticides) [10]. The cone snail toxin MVIIA is an analgesic drug targeting voltage-gated calcium channels (Ca_V_), which is used under the name Prialt^®^, to treat intractable pain [11]. Other toxins are also in development as treatments of various nervous system disorders (epilepsy, pain), as well as arthritis, heart and blood pressure disorders and cancer [12,13]. Considerable effort is undertaken to discover new toxins with interesting activity, and in the course of this research, extensive knowledge on how toxins are produced has been gained.

In most studied organisms, peptide toxins are stored in venom glands. The cells lining these glands produce toxins as precursor proteins, which undergo several maturation steps before being secreted [14]. Toxin precursors are first directed to the endoplasmic reticulum (ER), where the ER signal peptide region is cleaved. While transiting through the secretory pathway, the truncated toxin precursors mature by undergoing several post-translational modifications (PTMs), which include excision of the mature region, formation of disulfide bonds between pairs of cysteine residues and modifications of side chains or peptide termini, for example hydroxylation of prolines or amidation of the *C*-terminus [15,16,17]. The formation of disulfide bonds is a particularly important PTM because it stabilizes the three-dimensional (3D) structures of toxins, and consequently the number and arrangement of the disulfide bonds is commonly used to classify toxins [15]. (The many toxins that do not have disulfide bonds are typically classified in families according to their sequence similarities). Overall, 15 side-chain PTMs have so far been identified in cone snail toxins [15]. These modifications contribute to increasing the chemical diversity of venoms, fine-tuning the specificity for molecular targets or helping the maturation of toxins [18]. Studies of toxin evolution have revealed that most animal toxin genes belong to gene superfamilies that probably emerged from duplication of a few ancestral genes [19]. The ER signal peptide of toxin precursors shares high sequence similarity within a gene superfamily but the mature peptide regions undergo rapid evolution and are consequently more divergent [16]. This fundamental knowledge of toxin maturation and evolution has led to the development of targeted approaches to discover them.

**Figure 1:**
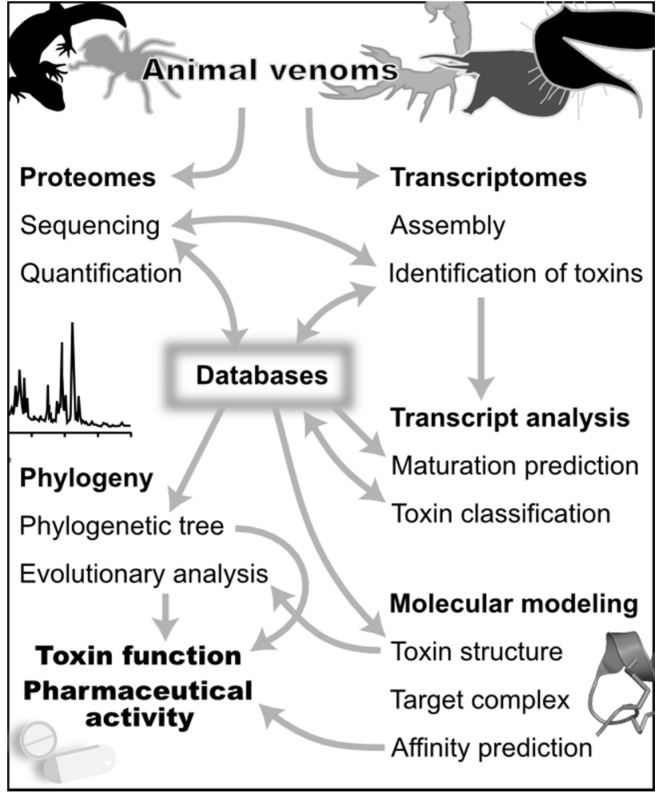
Computational approaches in venomics. The different themes in this figure are discussed in the text. Generalist and specialized databases are a central source of information for both the discovery (**top**) and analysis (**bottom**) of venoms. Venomics computational tools combine data from proteomics and transcriptomics to discover the set of toxins in a venom gland. These tools use predictions of mature toxins from transcriptomes to support peptide sequencing, and sequencing by mass spectrometry uses transcript sequences as a database to rapidly identify peptides and their post-translational modifications. Bioinformatics tools use the standardized classification stored in databases to analyze transcripts, and phylogenetic analyses can be used to analyze toxin evolutionary relationships. Databases in turn record the newly identified toxin peptide and transcript sequences. These sequence data can be also used to refine toxin classification, for example when a new phylogenetic group of toxins is identified. The molecular target of toxins can be suggested by a phylogeny and an evolutionary analysis complemented by the prediction of toxin 3D structures. Molecular modeling of target/toxin complexes can be used to analyze in great depth structure-activity relationships of toxins. Finally, the affinity of these complexes can be predicted from the molecular models.

Over the last few years, cutting-edge technologies are increasingly being employed in toxin research to unravel the pharmacological treasures hidden in animal venoms, dramatically changing the landscape of venom exploration [10]. Combined advances in proteomics and transcriptomics now give access to nearly complete toxin repertoires of a single venom [20], with this new approach termed “venomics” [2,10,21]. Venomics research is driven by techniques rather than by hypotheses [10], with old fractionation methods now replaced with the simultaneous discovery of massive amounts of toxin nucleic and protein sequences. This large amount of data cannot be efficiently comprehended without the use of computational and statistical methods [22]. This review will first focus on computational approaches that have been developed in recent years to help in the discovery and analysis of venom proteins and peptides. The main themes discussed are illustrated in Figure 1. Several specialized databases provide access to information on toxins, which are used by bioinformatics tools to analyze “omics” data and to orient new venomics research. The increased pace at which toxin sequences are being discovered is unfortunately not matched by the speed at which their functions are assayed because the functional characterization of a single toxin requires significant experimental efforts. Thus, we also describe some of the computational approaches that have been developed to suggest and study toxin functions.

## 2. Databases

Sequence and structure data on toxins are dispersed and difficult to extract from generalist sequence databases such as GenBank [23] because toxin sequences are not always annotated as such or because sequences are highly redundant [22]. Generalist databases rely on authors to submit their sequences, and consequently a large amount of information is in the peer-reviewed literature but not in generalist databases. Some aspects of toxin-centered databases were reviewed recently [24]. ConoServer (http://www.conoserver.org), ArachnoServer (http://www.arachnoserver.org), and ISOB (Indigenous Snake species Of Bangladesh, http://www.snakebd.com/) provide expert annotations on the sequences and 3D structures of cone snail, spider, and snake toxins, respectively [25,26,27]. There are currently no equivalent databases for other venomous species, and this absence has been noted as impeding venomics efforts [1]. ConoServer, ArachnoServer and ISOB catalog data that are either manually extracted from the peer-reviewed literature or semi-automatically retrieved from generalist sequence and 3D structure repositories such as UniProtKB [28], GenBank [23], GenPept and the Protein Data Bank (PDB) [29]. The origin of each entry in ConoServer, ArachnoServer and ISOB can be traced back to the peer-reviewed literature and/or to a generalist database. ConoServer and ArachnoServer databases solve naming issues related to toxins that have several names in the literature, ultimately giving names to toxins according to cone snail and spider toxin standard nomenclatures [15,30]. Another important task of these two databases is to keep track of the rapidly evolving toxin classifications, which are frequently amended. In the remainder of this section we now give more information on the five most useful publicly available sources of data in venomics, namely ConoServer [25,31], ArachnoServer [26,32], ISOB [27], Tox-Prot [33] and ATDB (Animal Toxin DataBase) [34].

ConoServer [25,31] catalogs 2201 mature toxin sequences, 2837 precursor sequences and 175 3D structures of cone snail toxins (as of June 2015), which have been retrieved from public repositories and from the peer-reviewed literature. Mature peptide toxin entries, protein and nucleic acid precursor entries, and 3D structure entries are linked. All mature peptide toxins, discovered experimentally or predicted from the precursor, are entered as independent entries. The prediction of mature peptide toxins allows identifying precursor sequences that mature into the same toxin, discovering these peptides by mass-spectrometry (MS), and aligning the mature peptides on the database website. The experimental evidence supporting the existence of each toxin, *i.e.*, if it was discovered at the protein or nucleotide level, is provided. ConoServer only records experimentally identified PTMs, and these are graphically annotated on the sequences of the mature toxins. The biological activity on molecular targets are described in terms of IC_50_, K_i_, and percentage of inhibition, and the targets are named according to the classification of the International Union of basic and clinical PHARmacology (IUPHAR) [35]. The function of a toxin can be queried using the “pharmacological family” classification, which describes the type of molecular target and the type of activity, for example the κ-conotoxins describe cone snail toxins that block voltage-gated potassium channels. NMR models are retrieved from the Biological Magnetic Resonance Bank (BMRB) [36] but some are also directly submitted to ConoServer by authors upon request. The database also keeps track of patented sequences as well as information on synthetic peptides derived from cone snail toxins; for example, synthesized for structure-activity relationship studies. Statistics on the classification schemes, PTMs, and toxins per cone snail species are automatically updated. Each entry has a stable identifier, which comprises the letter “P” for proteins or “N” for nucleic acids followed by a number. The peer-reviewed references that were used to create the database are listed on a single page, providing a convenient bibliography on cone snail toxin discovery. Database sequences can be retrieved in FASTA format, and all annotations can be downloaded in XML format. The database is regularly updated.

ArachnoServer [26] catalogs 1464 mature spider toxins, which were retrieved from UniProtKB [28] (as of June 2015). Mature toxin PTMs are provided and clearly identified as being experimentally validated or predicted by homology. Disulfide bond connectivities are difficult to determine experimentally, and ArachnoServer supplies an experimental confidence level for them. The toxin molecular targets are described according to the IUPHAR nomenclature [35], and the toxins can be browsed according to their molecular targets using a powerful interactive tool. ArachnoServer provides experimental values of target inhibition for a large number of entries, and considerable efforts have been made to provide comments on toxin functional characterization. Enzymes are described using the Enzyme Commission (EC) number and are linked to the MEROPS database [37]. All mature toxins in ArachnoServer are named according to the standard nomenclature for spider toxins [30], but alternative names found in the literature are also recorded. Each toxin entry has a unique identifier, which comprises the two letters “AS” followed by a number. Sequences can be retrieved in FASTA format, and every list of entries can be saved in XLS or PDF formats.

ISOB (http://www.snakebd.com/) [27] catalogs toxins from snakes in Bangladesh. This recently created database can be queried by the snake species scientific name as well as by toxins. The database interface does not allow searching toxins by name or sequence but they can be listed according to their activity, such as serine proteinase, neurotoxin or phospholipase. Information provided for each toxin includes its name, functional class, uniprot accession number, reference, protein sequence and structural information. When no experimental structure is available, a homology model is generated for each toxin. As of June 2015, 76 species of snakes and 419 proteins and toxins are listed in ISOB.

Tox-Prot (http://www.uniprot.org/program/toxins) [33] is the animal toxin annotation program of UniProtKB and aims to systematically annotate animal venom peptides [24]. Some of the annotations found in ConoServer and ArachnoServer have been transferred by Tox-Prot into UniProtKB, and links between the databases have been created. There are fewer annotated entries in UniProtKB concerning cone snail toxins than in ConoServer; for example only 1011 cone snail toxin sequences are annotated in UniProtKB. Each toxin entry in UniProtKB displays less annotation on the functional characterization than ArachnoServer or ConoServer. Besides cone snail and spider toxins, Tox-Prot stands as the best choice to retrieve information on toxins for the majority of other venomous species. Toxin activities are described in comment fields in UniProtKB entry cards. Two ontologies are used to describe ion channel and receptor subtypes: IUPHAR [35] and Human Genome Organization (HUGO) Gene Nomenclature Committee (HGNC) ontologies. Two other ontologies describe enzymes found in venoms: EC numbers and the nomenclature from the International Union of Biochemistry and Molecular Biology (IUBMB).

The animal toxin database (ATDB, http://protchem.hunnu.edu.cn/toxin/) [34] is a meta-database that displays information from several databases, including UniProtKB, GenPept and three discontinued databases dedicated to scorpion toxins (SCORPION2; [38]), cone snail toxins (MOLLUSK) and some snake toxins (svPLA2). ATDB is based on highly detailed ontologies describing the toxins and the target ion channels. ATDB toxin ontology describes the function of the toxins at different levels: The mode of actions and effects on the molecular targets, the symptoms, the pathogenesis (e.g., “Cytolysis/Cell degranulating/Muscle necrosis”) and the functional type of the toxins (e.g., “Neurotoxin/Postsynaptic neurotoxin”). The ion channel ontology describes the targeted ion channels according to the IUPHAR classification [35], distribution, physiology (e.g., “Fast deactivation”) and pharmacology (e.g., “System effects/Pain responses/Inflammatory pain”). These detailed ontologies would potentially allow very clever browsing of the database, but the interface currently fails to display the corresponding entries for the ion channel ontology. The database was last updated in 2009.

## 3. Discovery of Toxins from Venom Gland Transcriptomes

Because of the rapidly decreasing cost of sequencing nucleic acids, especially using massively parallel sequencing technologies, a large number of transcriptomic analyses of snake, cone snail, spider, scorpion and a few other animal venom glands have been carried out. Besides the discovery of pharmaceutically active compounds, these studies are also motivated by academic interests in the genetic mechanisms at the origin of toxin families [19] or in the co-evolutions of venoms and preys [39,40].

Only a few studies have reported the sequencing of the genomes of venomous species, which is a major undertaking because eukaryote genomes are typically large and have numerous tandem repeats. Currently, two snake genomes (Burmese python and king cobra) [41,42], two cone snail genomes (*Conus* *bullatus* and *C. consors*) [43,44], one scorpion genome [9], one spider genome [45], and the genomes of the honeybee [46] and parasitic wasps [47] were reported as being at various stages of completion. By contrast, a large number of studies have reported sequencing of the venom gland transcriptomes, which are more affordable and the bioinformatics analyses are easier than for sequencing genomes. Consequently, we focus here mainly on the analysis of transcriptomes rather than genomes.

The analysis of the transcriptome of a venom gland proceeds in several steps, which differ only slightly from the standard procedure for other tissues [48]: The sequencing reads are first assembled into contigs, the functions of contig gene products are predicted by homology, toxin precursors are identified among the contigs, toxin precursors are analyzed and classified, and reads and contigs are submitted to generalist repositories, such as NCBI SRA, dbEST and/or GenBank [49].

From a bioinformatics point of view, the important characteristics of the different sequencing technologies are the length of the reads compared to those of the transcripts, the number of reads, and the sequencing error rate. Second generation sequencing technologies, such as 454 GS FLX Titanium (454) and Illumina, give access to the sequences of lowly expressed transcripts that were undetected by Sanger sequencing methods [50], but the new technologies have a much higher rate of sequencing errors. For example, the 454 has an average error rate of 1% [51], and studies that claim observing transcript variations supported by only one sequencing read [52], should be viewed with caution. Newly discovered transcripts should ideally be supported by proteomics data or other nucleic acid sequencing technologies [50].

Sequencing reads are typically assembled *de novo* into larger contigs, and this procedure requires discarding low-quality reads. Illumina reads are typically first analyzed for their quality using the Illumina quality scores, and this analysis orients the choice of the quality threshold of reads as well as the trimming of low quality bases at the 3′ end of all reads. ESTs can be assembled into contigs using various software, the most popular being CAP3 [53], Phrap [54] and SeqMan [55]. The most popular assemblers for 454 reads are MIRA [56], Newbler (Life Science, Frederick, CO, USA) and CLC Genomics Workbench (CLC Bio, Aarhus, Denmark), and popular transcriptome assemblers used with Illumina reads are Velvet/Oases [57] and Trinity [58]. *De novo* assembly, *i.e.*, without a reference genome, of second-generation sequencing reads is still considered challenging and should be treated with caution. Multigene toxin families display high similarity in part of their sequences [16], and are therefore particularly difficult to assemble because similar or even identical read sequences and a certain rate of sequencing error are potentially shared between several transcripts. Issues associated with second-generation sequencing assemblies are the failure to assemble certain transcripts and the creation of spurious contigs (chimeric transcripts). Some studies assess the reliability of assemblies by cross validating results from several *de novo* assemblers [59], but such strategy is highly time consuming and not totally conclusive. The choice was made in some studies to avoid assembly problems and to search for toxin transcripts directly in the sequencing reads. The distribution of open reading frames of cone snail toxin transcripts is indeed similar to the distribution of read lengths produced by 454 sequencing technology [50,60]. VTBuilder is a recently developed transcriptome assembler that considers relationships between co-evolving sites to avoid building chimeric transcripts, and has been shown to be effective at assembling toxin transcripts from Illumina reads [61].

Contigs corresponding to toxin transcripts can be identified by similarity with known toxin sequences. The potential function of non-toxin contigs is predicted by homology with entries in NCBI/nr [49] and/or UniProtKB [28]. Standard annotations of non-toxin sequences include the enrichment in gene ontology terms using Blast2GO [62], and the identification of protein families, domains and important sites using InterProScan [63]. Proteins belonging to certain metabolic and regulatory pathways can also be identified using the KEGG annotation sever [64] and iPath explorer [65]. This approach was recently used to suggest that certain metabolic pathways are not directly involved with toxin production. For example, one study found that fatty acid synthesis is down-regulated in the venom gland of the cone snail *Conus geographus* [66].

Toxin precursors belonging to different gene superfamilies are highly divergent, and toxins belonging to previously undescribed gene superfamilies are difficult to identify using sequence similarity searches. Profile-based method alignments are more sensitive and have been used in a few studies analyzing venom gland transcriptomes [52,67,68]. For example, Shwartz *et al.* [68] used a sequence profile built with PSI-BLAST [69] to suggest the remote homology between toxin venom gland transcripts and other scorpion toxins that are potassium channel blockers. Profile-hidden Markov models (pHMMS) were recently used to identify toxin transcripts in cone snail transcriptomes [52,67,70,71]. ConoDictor uses pHMMs as well as generalized position-scoring matrices to classify cone snail toxin precursors into gene families or superfamilies [70,71], and this method has been used to identify toxins from transcriptomic data [72]. The sequence profiles of ConoDictor were constructed for signal peptide, pro-region and mature peptide region sorted into different groups based on sequence similarity. ConoDictor is an online tool (http://conco.ebc.ee), and it is therefore convenient to use. ConoSorter [52] uses several pHMMs to rapidly identify toxin transcripts in 454 reads or assembled contigs. Similarly to ConoDictor, the pHMMs of ConoSorter were created using HMMER [73], which was run on alignments of manually created clusters of transcript regions (signal peptide, pro-region and mature peptide region) for each gene superfamily. Alignment clusters were created manually because conventional clustering tools failed to produce reliable results, highlighting the extreme divergence between these sequences [52]. ConoSorter is a computer script, which needs to be run locally and can handle very large number of sequences. A simpler approach was recently employed by Robinson *et al.* [67]: One pHMM was created for each gene superfamily using alignments of all non-redundant sequences in ConoServer, without creating clusters [25]. Other approaches to identify toxins in assemblies or reads are based on the knowledge that toxins display a limited number of cysteine patterns in their amino acid sequence [59] or adopt a limited number of folds [74]. For example, giant ant *Dinoponera quadriceps* toxins were identified by local sequence alignment against the KNOTTIN database [75], which catalogs proteins adopting a cystine knot fold [76]. Knoter 1D is an on-line tool that automates this process by predicting cystine knot folds based on sequence similarity to known knotted proteins as well as a range of other features, such as the number of cysteine residues, the length of sequences between consecutive cysteine residues or annotation keywords [75]. Besides sharing similar folds, toxins acting at similar molecular targets should display similar sequence features, and ClanTox [77] is a machine learning approach that aims to identify ion channel inhibitors using sequence features only. The methods implemented in ClanTox combine the results of several decision-stump classifiers using the AdaBoost algorithm [78] (boosted-stump classifiers). ClanTox is species independent, as opposed to ConoDictor and ConoSorter, and can be used to identify toxin-like sequences. For example, ClanTox was used to discover toxin-like proteins in mammalian transcriptomes [79], and some of the identified peptides are modulators of cell signaling acting at nicotinic acetylcholine receptors using a three-finger snake toxin fold [80,81]. On the basis of convergent evolution of toxins in different phila, Starcevic *et al.* identified sequences motifs that seem to be specifically used by toxins [82]. They used MEME [83] to identify 748 shared motifs of about 40 amino acids in length, and the pHMM built from alignment of these motifs was shown to be able to identify snake PLA2 toxins among other proteins and to generate only a limited number of false positives when analyzing transcriptomes of non-venomous animals.

## 4. Discovery of Toxins from Proteomes

Most of the known toxin peptide sequences were predicted from conceptual translation of nucleic acid sequences. Consequently, the majority of toxins cataloged in databases do not have any experimental support for them being produced in venoms. For example, only 379 out of the 1873 mature toxins recorded in ConoServer have some experimental evidence of being present in venom. Nevertheless, support for mature peptide toxin sequences becomes more routinely and rapidly provided by modern proteomics experiments, e.g., [20,60]. A major issue in toxin proteomics derives from large intra-species and even intra-specimen variability, indicating that understanding the biology of the venomous animals is essential to characterize venom content [84,85]. Additionally, each venom displays a very large chemical diversity, which can be explained by recently evidenced mechanisms, including differential post-translational modification of toxins [86], differential truncations [60], and transcription messiness [50]. Transcription messiness is an emerging concept where errors of transcriptions cause epimutations, resulting in several proteins with slightly different amino acid sequences [87,88,89].

Proteomics approaches to analyze snake venom were recently reviewed [1,90], and the techniques discussed in these reviews are generally applicable to other animal venoms. Venoms typically contain hundreds of different peptide components [3], and a wide range of combination of chromatography, electrophoresis, enzymatic digestions, Edman degradation, or mass spectrometry (MS) techniques have been employed to sequence their toxins [1,90]. Edman degradation is the traditional approach to sequence peptides, and it still sometimes used for full [91] or partial [92] toxin sequencing. Most toxins are very small and can be sequenced at lower cost using tandem MS (MS/MS) [50,60,90], but Edman degradation can be useful as a complement to MS, e.g., reference [93], for example helping to identify the isobaric amino acids isoleucine/leucine and for *N*-terminal sequencing.

Peptide sequences are typically discovered from MS/MS data by (i) complete *de novo* sequence reconstruction, (ii) reconstruction of some partial sequence tags to be searched in a sequence database, or (iii) matching spectra predicted from a database of sequences. Complete *de novo* sequencing is slow and its challenging nature is exacerbated by the high frequency of side-chain PTMs. Despite the creation of some specific methodologies [94], this strategy is no longer used routinely. The identification of peptides in MS/MS spectra using proteins predicted from transcriptomes is now standard procedure [50,60,95]. This proteogenomic strategy leads to a higher level of confidence for the mature peptide toxin sequences because transcriptomes provide independent validation and also help to resolve ambiguities regarding the identification of isobaric residues, *i.e.*, isoleucine and leucine residues. The majority of side chain PTMs cannot be predicted from transcript sequences, and these PTMs potentially render the identification of peptides more difficult, but the widely used software for matching MS data with sequence databases, Mascot (Matrix Science, Boston, MA, USA), ProteinPilot (Applied Biosystems, Waltham, MA USA), SEQUEST (Thermo Finnigan, San Francisco, CA, USA), and x!tandem [96] readily take into account a very large number of PTMs. The latest developments in the field been have been recently reviewed [97,98], and we will focus on bioinformatics methods specifically made to analyze venom content. A tool called ConoMass, accessible from the ConoServer website, uses a brute force approach to match reconstructed peptide masses from MS data with toxin masses predicted from transcriptome analysis [25,60]. ConoMass considers all possible combinations of PTMs that were identified in cone snail toxins to date, with the exception of side chain glycosylations, which are very diverse in their occurrence [25,99]. We note that alternative disulfide bond connectivities, which can lead to variation in bioactivity [100], cannot be detected via mass changes. The identification of peptide using such a simple method, which is also employed by other algorithms such as FindMod [101], typically generates a large number of false positives [102]. Increased sensitivity of mass spectrometers as well as knowledge of PTMs commonly found in related peptides from the same gene superfamily could help to discriminate between alternative toxin sequences for each reconstructed mass [60].

The quantification of toxins in the venom can be carried out by integration of the area under chromatography elution curves. Interestingly, a recent comparison of the quantification from proteomes and transcriptomes in the venom gland of a snake showed large discrepancies, possibly because once the concentration of a toxin in the glands reaches a certain level, its production is repressed [103]. Some toxins predicted to exist from transcriptome data are not found in the proteome, and conversely toxins in the proteome have no identified transcripts. It is therefore important to cautiously interpret the expression level of transcripts discovered from a transcriptome analysis in terms of importance in the venom. Possible explanations of these discrepancies have been proposed [1]: Some toxins might not be secreted and will not be found in the venom; the origin of the material is different (several venom samples are typically pooled from several animals but RNA material is only collected from one specimen); the sensitivity of proteomics is still too low; and some types of side chain PTMs might still be unknown. An alternative explanation was proposed in a recent study of the origin of the ontogenic shift in venom content of the Central American rattlesnake [104]. miRNA levels were shown to be the main factor that modulates venom composition as the relative toxin transcriptional activity was similar whatever the development stage.

## 5. Bioinformatics Analysis of Toxin Transcripts and Classifications

The analysis of toxin transcripts typically proceeds by determining the different regions in the precursors (ER signal peptide, mature peptide region and pro-regions) as well as the estimation of their evolutionary relationships and the structural and functional class of their mature toxins. With the increasing number of known toxins, the majority of these tasks can be done by transferring annotations by homology. The ER signal peptide boundary is often predicted using SignalP [105], which is currently the best method to predict the presence of an ER signal peptide (Mathew correlation coefficient of 87%) but is less accurate at predicting the exact position of the cleavage (Mathew correlation coefficient of 68%) [105]. Using the knowledge that the ER signal peptide is highly conserved within gene superfamilies, an alignment of sequences can alternatively be used to predict the ER signal peptide cleavage site. The determination of the mature peptide region in the precursor sequence is far more complicated, because we still have limited knowledge on most proprotein convertases specific cleavage sites [106]. A “rule of thumb” that has been used in some studies is to assume that a cleavage after all basic residues, *i.e.*, arginine or lysine residues, surrounding the sequence region containing all cysteine residues, e.g., [107]. This simple strategy captures very approximately the cleavage sites of furin-like peptidases, leading to about 50% wrong predictions for Cone snail precursors of cysteine rich toxins, and cannot be used for toxins that do not have cysteine residues. A more rigorous approach was implemented in ProP, which is an artificial neural network trained to recognize proprotein convertase cleavage sites [108]. ProP is highly accurate at recognizing furin-like protease cleavage sites, with 89% balanced accuracy on a large dataset, but has only an average 61% balanced accuracy when considering all types of proprotein convertases. Only 113 out of 291, *i.e.*, 39%, of experimentally validated cleavages sites surrounding mature cone snail toxin regions correspond to furin-like protease cleavage sites, and therefore using ProP to annotate cone snail toxin transcripts leads to a significant number of wrong predictions. The number of furin-like cleavage sites in spider toxin transcripts is very low, and ProP achieved a sensitivity of 0% in a recent study focusing on spider toxins [109]. Two specific tools, ConoPrec [25] and SpiderP [109], have been developed to annotate cone snail and spider toxins, respectively.

ConoPrec is available on the ConoServer website. Cone snail toxin precursor sequences can be provided to ConoPrec either as nucleic acid or protein sequences. This tool first determines ER signal peptide region using SignalP and then mature peptide regions using a set of regular expression corresponding to proprotein convertase cleavage sites [106]. These regular expressions match cleavage sites of the well-studied furin proteases as well as cleavage sites from other protease families, the specificity of which were only suggested from statistical analysis of alignments of precursor sequences [106]. The protease at the origin of some of these cleavages have not yet been identified, such as for the “EtoR” cleavages, which do not exist in mammals [106]. Some cleavages seem to be specific of some species, for example the “LtoR” cleavages for which a cone snail proprotein convertase was isolated [110]. ConoPrec achieved ~80% balanced accuracy for the identification of the 291 verified cone snail toxin cleavage sites in ConoServer, and it was also recently benchmarked to have similar balanced accuracy for analyzing spider toxin precursors [109]. The ability to achieve high accuracy in analyzing precursors from animals other than cone snails indicates that ConoPrec could potentially be used generally for all animal venoms. A current limitation of ConoPrec is that it only predicts cleavage sites of disulfide-rich toxin precursors.

SpiderP is a recently developed tool that uses a support vector machine (SVM) to predict the location of proprotein convertase cleavage sites in spider toxin precursors [109]. The SVM of SpiderP uses Gaussian Radial Basis Function (RBF) kernels and was trained to recognize cleavage sites on an eight residue long window. A negative dataset of 8-mers was created using spider sequences that are not proprotein convertases. On an independent dataset of 46 spider toxin protein precursors, SpiderP achieved an impressive balanced accuracy of 90%. The absence of furin-like cleavage sites in spider precursors indicates that this tool will be less efficient at analyzing transcriptomes in animals other than spiders. The algorithm can be used on-line on the ArachnoServer website.

Cone snail toxins are classified (i) in terms of gene superfamilies; (ii) according to the pattern of their cysteine residues in the primary sequence, which partly reflects the 3D fold of the mature toxins; and (iii) using their pharmacological activity, often named with a Greek letter, e.g., α-toxins, are blockers of nicotinic acetylcholine receptors. Classifying newly discovered toxins is an important task, and ConoPrec classifies cone snail toxin precursors into cysteine frameworks using their pattern of cysteines, and it assigns precursors to a gene superfamily if their ER signal peptide is more than 90% identical to that of any member of known cone snail gene superfamilies [25]. ConoPrec also determines the closest cone snail toxin in the ConoServer database, and this homolog toxin could be used to suggest a pharmacological activity. Three PTMs can be predicted on the basis of specific sequence features, including pyroglutamylation of *N*-terminal glutamine, *C*-terminal amidation and γ-carboxylation of glutamates.

The conserved ER signal peptide sequence provides definitive support for classifying toxin transcripts into gene superfamilies. Bioinformatics tools have been developed to classify cone snail toxins into gene superfamilies from partial sequences missing the ER signal peptide. ConoDictor and ConoSorter [52,70,71], which were introduced in the section describing the identification of toxins from transcriptomic data, determine the closest toxin gene superfamilies using pHMMs and/or generalized position specific scoring matrices. [71] Other studies have applied machine-learning approaches to extract the mature toxin sequence patterns associated with each cone snail gene superfamily. For example, a strategy using pseudo-amino acid composition and multi-class SVM achieved 88% correct predictions [111]. The method called IDQD uses an algorithm based on Mahalanobis discriminant instead of a SVM and achieved a balanced accuracy of 90% as well [112]. Another study used diffusion maps to optimize prediction features, and achieved an overall accuracy of 90% [113]. A recent study used an original approach by considering the similarities between small subsequences of each sequence compared to the total pool subsequences [114]. This study used SVM as a classifier and achieved a balanced accuracy of 98%, according to the authors.

## 6. Phylogenies and Evolutionary Analysis

Transcriptome analyses are often complemented by the establishment of molecular phylogenies between related toxin transcripts. Because structure and function tend to be conserved through evolution, related toxins display similar properties and a gradation of traits is expected to be associated with increasing phylogenetic distances between toxins [115]. For example, snake toxins displaying a three-finger scaffold have taxon-specific activity, and toxins belonging to distantly related branches display different receptor subtype or target specificity [116]. Establishing the phylogeny of toxin sequences could therefore help in determining their function. Describing the evolution of venoms relative to prey has also been used to inform the discovery of new toxins with interesting pharmaceutical activity [117]; for example, by focusing on studying the venom of fish-hunting cone snails [118], or on clades whose venom pool remained so far unexplored [119].

Studying the evolution of toxin scaffolds brings some understanding on how folds evolved relative to their function. A recent study of scorpion toxins proposed that the Von Willebrand factor *C*-domain fold (SV-SVC) is the ancestral fold from which the inhibitory cystine knot fold (ICK) evolved, which in turn gave rise to the disulfide-directed β-hairpin fold (DDH) [39]. Scorpion ICKs and DDHs have both been extensively studied for their function, and DDH toxins are more potent on human ryanodine receptors than ICK toxins, indicating that selection pressure results in more active compounds [39]. The loss of cysteine residues 2 and 3 in a clade of snake three-finger toxins led to a greater potency on mammalian receptors, which correlated with change of diet and explosion of new toxins displaying this modification [116,120]. Hence, molecular phylogeny could help pharmacological investigations of animal toxins by suggesting the clades the most likely to contain pharmaceutically interesting compounds.

### 6.1. Building Phylogenetic Trees

Classical phylogenetic analyses of protein or nucleic sequences require the generation of a multiple sequence alignment, the accuracy of which has a critical influence on the resulting phylogeny. Toxin sequences are often substantially divergent and generating a reliable multiple sequence alignment is difficult. Some authors use sequence alignments generated by software such as ClustalW [121], which was used to establish the phylogeny of some spider toxins [122] or Muscle [123], which was used to study the evolutionary relationships between some cone snail toxins [124]. Other authors rely on manual alignments [125]. A common strategy to reduce noise due to regions with unreliable alignment is to delete them from the multiple alignments. In some studies, all position displaying with gaps are deleted [122], and in some others only positions above a percentage of gaps are removed [39,116]. In the study of Sunagar *et al.* [39], two alignments of ICK and DDH sequences, corresponding to two different evolutionary scenarios, were considered, and the main conclusions from the two resulting but slightly different phylogenies globally agreed.

The methods most frequently used to build phylogenetic trees are the neighbor-joining algorithm, maximum likelihood, maximum parsimony and Markov chain Monte Carlo Bayesian inference. MrBayes is [126] the most popular software for Bayesian inference. The Beast package can also be used for Bayesian inference [127]. Beast has a particular focus on calibrated phylogenies incorporating a time-scale [127], which was used to establish the phylogeny of four cone snail species based on the evolutionary history of gene duplications within a gene superfamily [19]. Maximum likelihood analyses of toxins were carried out using PhyML [128], PAUP* (Phylogenetic Analysis Using Parsimony) or MEGA (Molecular Evolutionary Genetics Analysis) [129]. MEGA was most often used for building maximum parsimony and neighbor joining trees in animal toxin studies. Substitution models were automatically selected using ModelGenerator [130], for protein and nucleic acid sequences, or ModelTest [131], for nucleic acid sequences only.

### 6.2. Evolutionary Analysis

Determining the positions under selective pressure could help in determining toxin functions. For example, positions under strong positive selections were shown to be important for selectivity and potency for some scorpion toxins [132]. CODEML of the PAML (Phylogenetic Analysis by Maximum Likelihood) package has often been employed to estimate several maximum-likelihood models that evaluate the ratio between non-synonymous to synonymous nucleotide substitution rates [133]. Another approach to compute this ratio is based on the Nei-Gojobori model [134], which is implemented in MEGA [129]. A ratio significantly greater than 1 indicates that the corresponding position is subject to positive selection (accelerated mutations, possibly for functional optimization); conversely, a ratio smaller than 1 indicates negative selection (slow mutation rates, possibly for maintaining function).

Fry and colleagues have employed Bayesian statistic methods to access better estimates of selective pressure on a range of toxin families [39,84,116] using BEB (Bayes Empirical Bayes) [135] and FUBAR (Fast, Unconstrained Bayesian AppRoximation) [136]. They also used an algorithm that works at the protein level called TreeSAAP [137] and a range of other algorithms to test episodic diversifying selection of toxins and taking into account the influence of recombination events [39,116].

## 7. Prediction of Toxin Structures and Activities

The functional characterization of toxins requires significant experimental effort, and consequently the majority of known toxins have not been experimentally tested for activity. A common assumption is that toxins belonging to the same gene superfamily and displaying the same cysteine framework will have similar targets. Whereas this approach often proves to be correct, some toxins sharing high sequence similarity have also shown different specificities. For example the spider toxins κ-theraphotoxin-Ps1b and β/ω-theraphotoxin-Tp2a share 77% sequence identity, but κ-theraphotoxin-Ps1b acts on voltage-gated potassium (K_V_) and mechanosensitive channels [138,139], whereas β/ω-theraphotoxin-Tp2a is only active on voltage-gated sodium (Na_V_) and Ca_V_ channels [140,141]. This section describes computational methods that could help assign or study toxin activity.

Toxins seem to adopt a limited number of structural scaffolds and it was initially hypothesized that the target selectivity of toxins could be predicted from statistical analysis of toxin sequences for each scaffold [22]. An algorithm based on neighbor-joining clustering with annotated scorpion toxins precursors was designed to predict ion channel targets, *i.e.*, specificity for Na_V_, K_V_, Ca_V_ or chloride channels [142], but this approach only resulted in 69% specificity. A more recent algorithm based on RBF network, which is a type of artificial neural network, achieved 89% accuracy at predicting the ion channel targeted by cone snail toxins [143]. This study used the frequency of di-peptide as features, and the most significant di-peptide sequences were selected based on the binomial distribution of their frequencies [144]. This approach offers hope that such bioinformatics approaches could accurately predict the type of targets of each animal toxin. Nevertheless, the prediction of toxin selectivity at the subtype level, which is the characteristic that generates most excitement about toxins, seems beyond reach using sequence information only. Indeed, a given toxin typically has a wide dynamic range of affinities, from picomolar to micromolar, on distinct molecular targets, tremendously complicating the prediction of toxin specificity through the sole use of amino acid sequence information. Predicting specificity for receptor subtypes requires detailed description of toxin molecular structures and interactions.

### 7.1. Molecular Modeling of Toxin Structures

Toxin structure-activity relationships studies aim to determine the structural features important for toxin target activity. Such studies proceed by comparing the 3D structures of wild-type and modified peptides to discover shared structural motifs that could be linked to function [115,122]. For example, cystine knot spider toxins that interact with the voltage sensors of ion channels display an important hydrophobic loop between their first two cystine residues [145] and this knowledge was used to suggest the function of newly discovered toxins [122]. Other structural features measured on structures are the accessible surface area of each residue and the electrostatic potential created by the toxins in a water environment, which could be computed by solving the Poisson–Boltzmann equation. The 3D structures of a large number of toxins have been solved by nuclear magnetic resonance (NMR), and these structures serve as templates to build homology models [39,116,122,146]. Most homology models were built using Modeller [147] or Swiss-Model (http://swissmodel.expasy.org/). In the absence of homologous toxins with experimentally determined structures, some studies attempted *ab initio* structural modeling. For example, the I-TASSER (Iterative Threading ASSEmbly Refinement) server [148] was used to model the scorpion toxin MeuTXK(beta)5-NHD(S) [115], and the PEP-FOLD server [149] was recently used to model the structure of cone snail toxin Vt3.1 [150]. I-TASSER provides a confidence score that can be used to assess if toxins at least adopt similar folds, and this score was used recently to suggest the fold adopted by three box jellyfish toxins [151]. In any modeling approach, it is important to remember that any given structural motif can be displayed by proteins having different activities; for example, the cystine knot scaffold is used by some ω-conotoxins to block calcium channels [152]; some κ-conotoxins to block potassium channels [153]; and some spider toxins to block sodium channels [154].

### 7.2. Molecular Modeling of Complexes with Molecular Targets

The molecular targets of toxins are typically very large transmembrane proteins, and there is currently only one crystallographic structure of a toxin in complex with its real molecular target: The tarantula psalmotoxin bound to a chicken acid-sensing ion channel [155]. Some structures of complexes between toxins and some structural surrogates have been determined experimentally [156]. Indeed, some cone snail and snake toxins have been co-crystallized with mollusk acetylcholine-binding proteins (AChBPs), which are structural homologs of the ligand-binding domain of nicotinic acetylcholine receptors (nAChRs) [157]. Molecular modeling is routinely used to gain insights on the molecular interactions at the origin of toxin affinity and specificity. The first step of this process is the structural modeling of the molecular targets, often performed by homology modeling based on some key experimental structures, including the crystal structure of rat K_V_1.2 [158], the crystal structure of bacterial sodium channel Na_V_AB [159], the electron microscopy structure of torpedo nAChR [160], the crystal structures of AChBPs [157] or the crystal structure of chicken ASIC1 channel [161]. Toxins often bind to a specific state of their molecular target, and the modeling of this state could be particularly difficult, especially if no relevant structural template is available, e.g., toxins binding to specific states of voltage-gated ion channels.

A determination of the binding mode of toxins binding to nAChR subtypes can confidently be modeled by homology on the basis of the conserved binding modes of homologs crystallized in complex with AChBP [162,163,164,165,166,167]. Some toxins are protease inhibitors and the modeling of their complexes, e.g., reference [168], can also be made confidently because of the large number of crystallographic structures of proteases. In the absence of structural homologs, molecular docking and different types of molecular dynamics (MD) simulations have been employed, as recently reviewed in detail [169]. The accuracy of such approaches greatly benefits from additional experimental data on the interaction, e.g., mutational data on both toxins and receptors, cross-linking experiments or interaction studies by NMR. Docking methods combine theoretical and heuristic techniques to produce a large number of docked “poses”, which are then clustered and ranked on the basis of a scoring function or using knowledge from experiments. The docking software Autodock [170], Gold (Statistical Innovations Inc., Belmont, MA, USA), Hex, ZDOCK [171] and HADDOCK [172] are among the docking algorithms the most frequently used for toxin binding studies. The ZDOCK scoring function allows some structural overlap at the interface, accounting for conformational variations at the interface. This method was successfully used to predict the interaction of mambalgin-2 toxin with ASIC1 channels, the fuzziness of the docking procedure allowing docking to the desensitized state even if the toxins is known to preferentially bind the closed state of the channel [173].

All-atom MD simulations are frequently employed to refine the conformation of models of molecular complex resulting from docking as well as to discover the binding modes of toxins. Because toxin/target systems typically have more than a hundred thousand atoms, the simulation times are limited to 10–100 ns, which is too short to observe spontaneous binding [169], except in some rare cases [174]. The use of biasing potentials (for example, harmonic restraints to maintain the toxin at proximity of the hypothesized binding site) allowed several studies to successfully propose some toxin binding modes [174,175]. An interesting and recent study used coarse-grained (CG) MD simulations instead of the standard all-atom MD to simulate the interactions between the spider toxin HaTx1 and a human K_V_2.1 [176]. During a 3 μs un-biased simulation, the HaTx1 first embedded in the phospholipid membrane bilayer and then interacted with the voltage-sensing domain of the channel [176]. CG representation substantially reduces the number of atoms in the simulated system, and simulations using CG force fields can consequently reach considerably longer time scales than all-atom MD simulations. Brownian dynamics is another type of simulation that has been used to predict the binding mode of several toxins without imposing any arbitrary restraints, and this technique was reviewed recently [169]. In this approach, the molecules are maintained partially rigid and water molecules are represented implicitly using the Generalized Born model, dramatically decreasing the number of atoms in the system [177]. Yu *et al.* [178] used Brownian dynamics to suggest that the six toxins AgTx2, ChTx, KTx, MgTx, NTx, and Pi2 have similar binding modes when bound to K_V_1.3. The binding modes discovered by docking and MD could be experimentally validated by testing the activity of toxin variants, as suggested from the molecular models [163]. Another common approach to assess the validity of the resulting molecular model is to evaluate if it could be used to qualitatively explain mutation studies [164,167,174] or to reproduce absolute binding energies or relative mutational energies that have been measured experimentally [162,163,178].

### 7.3. Prediction of Toxin Binding Affinities and Specificities

As mentioned previously, the current major bottleneck in toxin research is the determination of the specificity of individual toxins, and several computational approaches that use extensive MD simulations to predict binding affinity could potentially help solve this problem. The most accurate method to quantitatively predict the binding affinity between peptides and proteins is the computation of potential of mean force (PMF) along unbinding paths sampled using umbrella sampling MD simulations [179]. Two recent reviews present, in detail, the use of this technique for studying the binding of animal toxins to ion channels [169,180]. The umbrella sampling procedure consists of carrying out a series of MD simulations starting at different coordinates along the unbinding pathway. The average energy as a function of the reaction coordinate, *i.e.*, the PMF, is then computed for each simulation, and the PMFs are finally combined into one using the weighted histogram analysis method (WHAM) [181]. In a recent study using this technique, the predicted dissociation constants of the toxins Css4 and Cn2 from a Na_V_ were 20 nM and 70 nM, which are comparable to the experimental dissociation constants of 1 nM and 40 nM, respectively [182]. In general, the dissociation constants predicted using the PMF/umbrella sampling method are within one or two orders of magnitude of experimental values [169]. An alternative technique used to build the PMF consists of using the Jarzynski equation to analyze a simulation in which unbinding pathways are rapidly sampled using a pulling potential [183]. When applied to a system comprising a toxin and a potassium channel, the method was found to be less sensitive and required more simulations to converge than the weighted histogram method [184]. Another study that focused on a complex between conotoxin ImI and a nAChR used a slower pulling speed and obtained at reasonable computational cost a one fold difference between the predicted (−70 kcal/moL) and experimental (−9 kcal/mol) binding free energies [164].

The PMF-based methods are computationally intensive, and other less accurate methods need to be employed to rapidly screen large libraries of toxins. One popular approximation requires sampling the conformational ensembles in the unbound and bound states only, and the binding energy is approximated by the molecular mechanics/Poisson–Boltzmann surface area (MM/PBSA) function [185]. This method was employed to study a system comprising conotoxin ImI and nAChR α7, and the predicted mutational energies of 16 ImI variants correlated with experimental values with a *R*^2^ correlation coefficient of 0.74, which is considered as accurate [162]. MM/PBSA was also successfully employed in several other *in silico* mutational studies of toxin/target systems [163,186]. For example, it was recently used to determine the binding sites of the analgesic conotoxin Vc1.1 on the α9α10 nAChR subtype, quantitatively predicting the impact of mutations that were later used to validate the binding mode experimentally [163]. The MM/PBSA method has a low computational cost but can potentially produce large errors depending of the simulation protocol [187]. We anticipate that this type of approach could be used to predict the subtype specificity of animal toxins, even if the accurate predictions of binding affinities are beyond its scope.

## 8. Future Perspective: Integrating the Biology of Venoms and Prediction of Toxin Activity

Bioinformatics is important at every step of venomics-based discovery. Databases orient venomics research by providing a concise overview of current knowledge on toxins, highlighting the venomous species or the type of toxins that have higher potential to become drugs, and suggesting what venomics fields are still terra incognita. In recent years, specific approaches have been invented to mine combined data from transcriptomics and proteomics, dramatically accelerating the pace of toxin discovery. These new approaches trade accuracy for speed, and we expect future improvements of the quality of experimental data as well as of the reliability assessment of results produced by venomics specific software. Our knowledge of venoms has rapidly expanded, and our estimate of the number of toxins has increased even more rapidly. We indeed now realize that venom biology is important and complex, as exemplified by some observations of extreme intra-specimen venom variability. We now also comprehend the complexity of venom chemistry, especially since it is now clear that venoms contain many more peptide toxins than there are genes encoding toxins in the genome. Currently, the description of these types of diversities is not well managed by venomics databases and bioinformatics tools. The aim of venomics efforts is to rapidly discover peptides with pharmaceutical activity, but paradoxically the venomics “pipeline” does not incorporate any new experimental approach to rapidly assess peptide functions. Computational approaches to predict the function of toxins already exist but applying them to the study of all toxins is not practical. Indeed, the most accurate computational methods to predict toxin affinities require a computation time higher than measuring activity by electrophysiology. Venomics is in great need of new molecular modeling and bioinformatics tools able to suggest the molecular target specificity of toxins.
